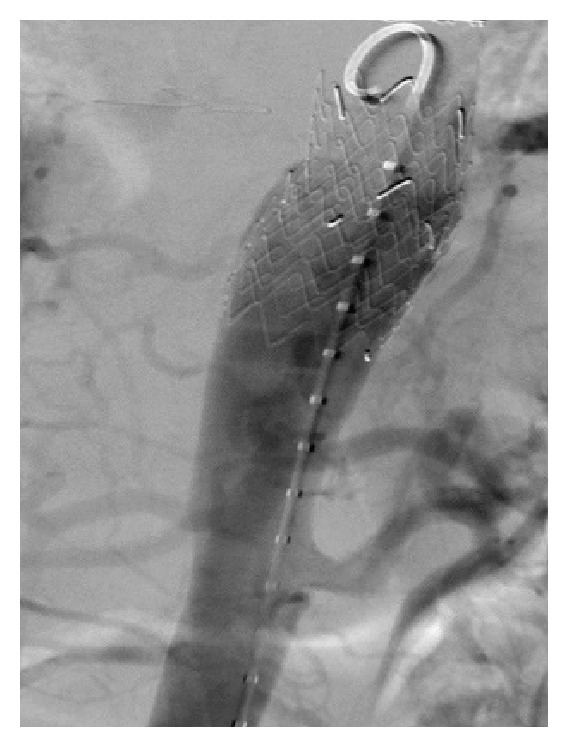# A Case of Glucocorticoid Remediable Aldosteronism and Thoracoabdominal Aneurysms

**DOI:** 10.1155/2016/2017571

**Published:** 2016-06-05

**Authors:** Anahita Shahrrava, Sunnan Moinuddin, Prajwal Boddu, Rohan Shah

**Affiliations:** ^1^Department of Internal Medicine, Advocate Illinois Masonic Medical Center, 836 West Wellington Avenue, Chicago, IL 60657, USA; ^2^Department of Radiology, Advocate Illinois Masonic Medical Center, 836 West Wellington Avenue, Chicago, IL 60657, USA

## Abstract

Glucocorticoid remediable aldosteronism (GRA) is rare familial form of primary aldosteronism characterized by a normalization of hypertension with the administration of glucocorticoids. We present a case of GRA and thoracoabdominal aneurysm complicated by multiple aortic dissections requiring complex surgical and endovascular repairs. Registry studies have shown a high rate of intracranial aneurysms in GRA patients with high case fatality rates. The association of thoracoabdominal aneurysms with GRA has not been described, thus far, in literature. Studies have shown that high tissue aldosterone levels concomitant with salt intake have a significant role in the pathogenesis of aneurysms and this may explain the formation of aneurysms in the intracranial vasculature and aorta. The association of GRA with thoracic aortic aneurysms needs to be further studied to develop screening recommendations for early identification and optimal treatment. Also, the early use of mineralocorticoid antagonists may have a significant preventive and attenuating effect in aneurysm formation, an association which needs to be confirmed in future studies.

## 1. Introduction

Glucocorticoid remediable aldosteronism is rare familial form of primary aldosteronism characterized by a unique clinical response of hypertension and aldosterone production to the administration of glucocorticoids. First described in 1996 by Sutherland and colleagues in a family of father and son, it was observed that the clinical findings of mineralocorticoid excess including hypertension and hypokalemia reversed dramatically with administration of dexamethasone giving it the name dexamethasone remediable hyperaldosteronism, also referred to as Familial Hyperaldosteronism Type 1 [1]. GRA has been associated with early onset familial intracranial aneurysms, a potentially fatal complication carrying high fatality rates. The association of GRA with thoracoabdominal aneurysms has not been studied. Studies suggest that high aldosterone levels and high salt intake have a significant hypertension-independent effect in the pathogenesis of aneurysms [2].

## 2. Case Report

A 24-year-old male presented to our hospital with daily complaints of chest pain and palpitations for the past three months. He endorsed to not being compliant with his prednisone and antihypertensives in the recent past. His medical history was significant for glucocorticoid remediable hyperaldosteronism (GRA) diagnosed at the age of 18, HTN, depression, and anxiety. His cardiovascular history was notable for 3 aortic dissections, at ages of 10, 17, and 22, mid-thoracic aortic aneurysm requiring endovascular repair at the age of 18, and abdominal aortic aneurysm which required open surgical repair at the age of 22. Childhood history was remarkable for early onset hypertension discovered at the age of 10 after his first episode of dissection. He also had severe headaches during childhood which were attributed to hypertension by his treating physicians. He was started then on amlodipine, carvedilol, and clonidine for the management of his hypertension. 8 years later, he was diagnosed with GRA by another physician and was started on prednisone and spironolactone. The biochemical data could not be obtained as it was done many years ago at an outside hospital, but diagnosis was confirmed by detecting the chimeric gene via PCR testing. Genetic testing was done on parents and the father was found to have abnormal gene. No family history of aortic aneurysms and negative gene testing ruled out familial thoracic aortic aneurysm and dissection. The patient's mother and father were not consanguineous, and both parents died of drug overdose. The patient's sister has Crohn's disease. The patient had a 4 pack-year smoking history.

**Figure 1:**
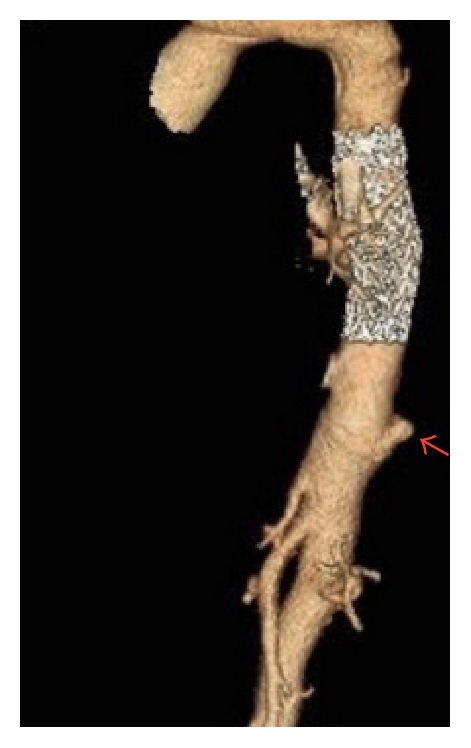


**Figure 2:**
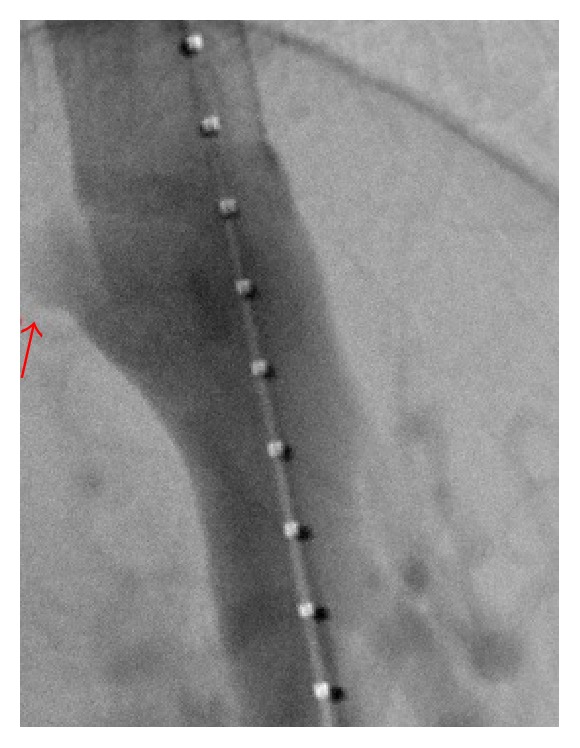


On initial exam, patient was hypertensive with a blood pressure of 206/109. Physical exam was unremarkable except for a 4/6 systolic murmur most prominent in the aortic area with radiation to the apex. The patient did not have morphological features of related conditions like Marfan's syndrome, Ehlers-Danlos syndrome, or Loeys-Dietz syndrome. Labs either were within normal limits or were unremarkable. Transthoracic echocardiogram revealed concentric hypertrophy with an ejection fraction of 60% and an intimal flap suggestive of aortic dissection. There was no evidence of cardiac anomalies of bicuspid valve on echocardiograph. CT angiogram (Figure 1) demonstrated chronic dissection of the aortic arch (not shown in figure) terminating at thoracic stent and extending into the innominate and common carotid arteries and a pseudoaneurysm of the distal thoracic aorta just above the celiac artery (Figures 1 and 2). The patient was started on antihypertensives and prednisone with gradual improvement of blood pressure back to baseline. Review of past medical records confirms that the aortic dissection was chronic and it was decided not to operate upon the patient due to high risk of surgical complications. Interventional radiology was consulted and an endovascular repair of the pseudoaneurysm was planned. An endovascular aortic stent was placed successfully without complications (Figures 2 and 3) and the patient was transferred to ICU for close monitoring.

## 3. Discussion

Glucocorticoid remediable aldosteronism is a rare form of familial hyperaldosteronism characterized by an autosomal dominant pattern of inheritance [3]. GRA is the most common monogenetic form of hypertension. Molecular studies have characterized the genetic basis of GRA to be from the unequal crossing over between CYP11B1 (11*β*-hydroxylase) and CYP11B2 (aldosterone synthase) loci resulting in chimeric gene involving the 5′ ACTH-responsive promoter of the 11*β*-hydroxylase gene to the 3′ coding sequences of the aldosterone synthase [4, 5]. This results in ectopic expression of aldosterone synthase in zona fasciculata under the modulation of ACTH resulting in ACTH mediated aldosteronism [4, 6].

GRA is characterized by early onset severe hypertension starting in early childhood with up to 80% of the affected presenting before the age of 13 [7]. However, associated studies have observed a large variation in the expression of phenotype among affected family members with some having only mild hypertension and others being normotensive [8]. Most patients with GRA are normokalemic in salt restricted state making potassium levels an insensitive tool for evaluating GRA indicating PHA [9, 10]. GRA is a low renin hypertension characterized by high aldosterone/renin ratio, failure to suppress aldosterone with salt loading, and elevated 18-hydroxycortisol, 18-hydroxycorticosterone, and 18-oxocortisol levels [11, 12]. However, definitive diagnosis is best accomplished by genetic testing for the chimeric gene by PCR in the peripheral blood DNA [13]. Physicians should maintain a high degree of suspicion for GRA in children with early onset severe hypertension especially with a supporting family history of early onset hypertension [7].

Early cerebrovascular complications in GRA were systemically reviewed in a cohort of 376 patients from 27 GRA pedigrees which showed the presence of intracranial aneurysms in 48% of all GRA pedigrees and case fatality rates of up to 61% [14] leading to screening recommendations for intracranial aneurysms every 5 years after puberty [15]. However, the incidence of thoracoabdominal aneurysms in GRA has not been studied to date. Mineralocorticoid receptors are expressed not only in the kidneys but also in the heart and the aorta [16]. It has been proven that aldosterone exerts widespread cardiovascular effects including left ventricular hypertrophy, hypertension, and heart failure independent of changes in systemic blood pressure indicating a potential remodeling role for mineralocorticoid antagonists [17]. A case report of successful treatment of a pseudoaneurysm in a type 2 diabetes mellitus patient while treating primary aldosteronism with spironolactone has been described [18]. Mouse models of aortic aneurysms have identified a significant role of aldosterone in the pathogenesis of aortic aneurysms. High aldosterone concomitant with increased salt intake leads to age-dependent aneurysmal changes in the aorta which do not correlate with blood pressure increases and reduce in size with mineralocorticoid receptor antagonists like spironolactone [2]. The results of this study lend to the proposal that early use of mineralocorticoid antagonists may have a significant preventative and remodeling effect of aneurysm formation in GRA patients and that early diagnosis of GRA remains pivotal to allow for prompt screening and early initiation of these agents. Also, case studies of aortic dissection in hyperaldosteronism suggest that high aldosterone levels may exert structural alterations in the aorta beyond and independent of aldosteronism-induced hypertension [19, 20]. The combined risk factor profile of hypertension, smoking, and hyperaldosteronism may well explain early onset of dissection and aneurysms in our patient.

The first-line treatment of GRA is the nightly use of dexamethasone or prednisone in doses sufficient to suppress early morning surges in ACTH and normalize blood pressure [21]. The initiation of mineralocorticoid antagonists in the treatment regimen is less clear and is generally considered in patients whose blood pressure is not normalized on glucocorticoids or if there is coexisting essential hypertension [22]. As discussed above, early use of mineralocorticoid antagonists may have far reaching benefits in preventing and/or attenuating aneurysm formation and should be considered early in the course of therapy even in normotensives.

To our knowledge, thoracoabdominal aneurysms in GRA have not been described in literature. The association of GRA with thoracic aortic aneurysms needs to be further studied to inform screening recommendations for early detection and optimal management of aortic aneurysms in these select groups of patients. The early use of mineralocorticoid antagonists may have a significant preventive and attenuating effect in aneurysm formation, an effect which needs to be confirmed in future studies.

## Figures and Tables

**Figure 3:**